# Protective Effect of Neochlorogenic Acid on Diabetic Nephropathy via Inflammation and Pyroptosis Suppression

**DOI:** 10.1021/acs.jafc.5c09087

**Published:** 2025-10-08

**Authors:** I-Ning Tsai, Yung-Che Tsai, Hui-Pei Huang, Ching-Chun Chen, Tung-Wei Hung, Chau-Jong Wang

**Affiliations:** † Institute of Medicine, 34899 Chung Shan Medical University, Taichung 402, Taiwan; ‡ Department of Nutrition, Chung Shan Medical University, Taichung 402, Taiwan; § Department of Medical Research, 63276 Chung Shan Medical University Hospital, Taichung 402, Taiwan; ∥ Department of Biochemistry, School of Medicine, Chung Shan Medical University, Taichung 40242, Taiwan; ⊥ School of Medicine, Chung Shan Medical University, Taichung 402, Taiwan; # Department of Medicine, Division of Nephrology, Chung Shan Medical University Hospital, Taichung 402, Taiwan; ∇ Department of Health Diet and Industry Management, Chung Shan Medical University, Taichung 402, Taiwan; ○ Department of Medical Research, Chung Shan Medical University Hospital, Taichung 402, Taiwan

**Keywords:** diabetic nephropathy, neochlorogenic acid, pyroptosis, miRNA regulation, inflammation, Nrf2 signaling, NF-κB

## Abstract

Diabetic nephropathy (DN) is one of the leading causes of end-stage renal disease. Neochlorogenic acid (nCGA), a polyphenolic compound present in various plant-based foods, has been recognized for its potent antioxidant and anti-inflammatory properties. However, its mechanistic effects on pyroptosis and microRNA signaling in the context of DN remain unclear. We investigated the molecular effects of nCGA on oxidative stress, inflammation, pyroptosis, and miRNA regulation in MES-13 mouse mesangial cells. nCGA enhanced Nrf2 activation, upregulated antioxidant genes, and suppressed ROS accumulation. It inhibited NF-κB signaling and reduced inflammatory proteins and also attenuated pyroptosis by downregulating NLRP3, GSDMD, caspase-1, and IL-1β. MicroRNA profiling revealed dual regulation: nCGA upregulated miR-30a, suppressing NF-κB/TNF-α pathways, and downregulated miR-709, thereby restoring the Nrf2-mediated antioxidant defense. Transfection with miR-30a mimics or miR-709 inhibitors reproduced these protective effects. These findings highlight the dual miRNA-mediated regulation, providing novel therapeutic insights into mitigating oxidative stress and inflammation in diabetic nephropathy.

## Graphical abstract

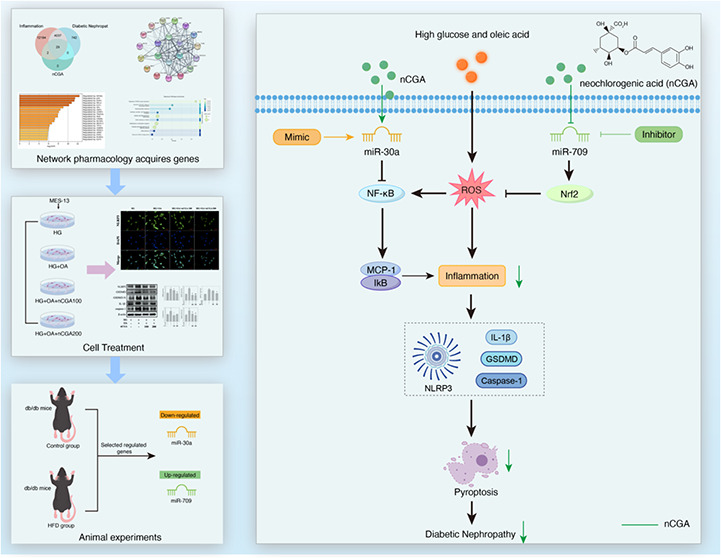

## 1 Introduction

Diabetic nephropathy (DN), one of the most common and severe complications of diabetes mellitus (DM), is a chronic kidney disease caused by metabolic disorders such as hyperglycemia and hyperlipidemia, which are associated with diabetes.^1^ Diabetes and DN have multifaceted mechanisms, involving a combination of hemodynamic and metabolic processes, oxidative damage, and the release of cytokines and growth factors, which ultimately result in kidney damage.^2^ Renal filtration function depends on the physical structure of the kidneys, which extract metabolites from the blood through ultrafiltration. Diabetes can damage renal tissues through mechanisms such as inflammation, leading to alterations such as mesangial expansion or thickening of the glomerular basement membrane, and these alterations may even result in nephrogenic systemic fibrosis.^3^ One of the major side effects of DN is kidney fibrosis, also termed mesangial expansion. This structural lesion is characterized by abnormal proliferation of mesangial cells and increased production of matrix proteins. In patients with DN, mesangial expansion substantially contributes to the progression of kidney failure,^4^ although the underlying causes and mechanisms remain unclear.

Reactive oxygen species (ROS) play a key role in the pathogenesis of diabetic complications. In patients with diabetes, increased oxidative stress directly results from the hyperglycemia-induced overproduction of ROS.^5^ Various vascular cell types, including renal cells, can generate ROS under hyperglycemic conditions.^6^ Under hyperglycemic conditions, ROS regulate the activation of signal transduction cascades and transcription factors, resulting in the transcriptional activation of profibrotic genes within the kidneys. Both conventional and catalytic antioxidants are effective in preventing or delaying DN.^7^ Previous research has elucidated a hierarchical oxidative stress model, in which varying levels of oxidative stress have distinct biological effects. At low levels, oxidative stress induces Nrf2, a transcription factor involved in the transactivation of genes encoding antioxidant enzymes. At intermediate levels, ROS initiate an inflammatory response through the activation of the transcription factor NF-κB.^8^

Keap1-Nrf2-ARE signaling is a well-characterized regulatory pathway that plays a crucial role in maintaining cellular redox equilibrium and protecting cells against various internal and external stressors. In this signaling pathway, ROS serve as key mediators, facilitating a dynamic equilibrium between the activation of Nrf2 and its inhibition by Keap1. This interaction underscores the role of ROS in modulating cellular defenses against oxidative damage.^9^ Under physiological conditions, Keap1 maintains Nrf2 at low concentrations in the cellular environment. Nevertheless, high levels of ROS lead to the oxidation of specific cysteine residues on Keap1, hindering its ability to promote the degradation of Nrf2.^10^

TNF-α is excessively produced in the muscles and adipose tissues of individuals with obesity. Lipotoxicity-induced inflammation plays a critical role in diabetic nephropathy. TNF-α is a key mediator produced by adipose tissue in obesity, contributing to both systemic and renal inflammation.^11^ A study involving 160 patients with type 2 diabetes^12^ reported an independent correlation between urinary TNF-α excretion and the clinical markers of DN.

Recent evidence suggests that pyroptosis, a form of proinflammatory cell death characterized by gasdermin D (GSDMD)-mediated pore formation in the plasma membrane, cellular swelling, rapid lysis, and the release of proinflammatory factors such as interleukin-1β (IL-1β) and interleukin-18 (IL-18), may play a significant role in the pathogenesis of DN.^13^

MicroRNAs (miRNAs) are small RNA molecules composed of approximately 22 nucleotides. These molecules function as post-transcriptional regulators of gene expression, influencing a range of biological processes, such as apoptosis, cell proliferation, and immune responses.^14^ Multiple studies have indicated the importance of miRNA-based therapy for DN. A review study^15^ explored the potential associations between miRNA activity in renal cell signaling pathways and DN progression. In their study, they emphasized the importance of mammalian targets of rapamycin, transforming growth factor-β, and phosphoinositide 3-kinase–protein kinase B pathways in the development of DN. Other studies have reported that miRNAs provide valuable insights for understanding disease mechanisms and diagnosing conditions, particularly in kidney pathologies, for which they offer new molecular targets. The previous study established a link between miR-709 expression in renal proximal tubular cells (PTCs) and acute tubular injury.^16^ They indicated that miR-709 had an influence on mitochondrial function, suggesting its potential as both a biomarker and a molecular target in renal diseases.

Neochlorogenic acid (nCGA), an isomer of chlorogenic acid (CGA), is a prominent phenolic compound abundant in plant-based diets and a key constituent in several traditional Chinese medicine formulations.^17^ Both chlorogenic acid (CGA) and neochlorogenic acid (nCGA) belong to the family of caffeoylquinic acid isomers, but their structural differences lie in the position of the esterified caffeoyl group on the quinic acid backbone. CGA is predominantly 5-*O*-caffeoylquinic acid, whereas nCGA is the 3-*O*-caffeoylquinic acid isomer. Recent comparative metabolism research further revealed that CGA generated a broader spectrum of metabolites (50 identified) in vivo, while nCGA produced relatively fewer (43 identified), reflecting differences in their metabolic stability and transformation pathways. Moreover, although both compounds and their metabolites shared common anti-inflammatory processes and signaling pathways, network pharmacology analyses demonstrated subtle differences in their biological targets, indicating that nCGA may exert distinct pharmacological actions compared with CGA.^18^ Previous studies have indicated that CGA can inhibit NLRP3 inflammasome activation and alleviate oxidative stress via activation of the Nrf2 signaling pathway, thereby providing renoprotective effects in diabetic nephropathy (DN) models.^19^ Furthermore, accumulating evidence suggests that CGA possesses broad pharmacological activities, including anti-inflammatory, antioxidant, antifibrotic, and metabolic regulatory effects—across multiple organ systems, including the kidney.^20^

Building upon our previous findings that mulberry leaf extract enriched with nCGA significantly ameliorates glucolipotoxicity-induced DN in HFD-fed db/db mice—through improvement of lipid metabolism, renal structure, and modulation of key signaling pathways such as JAK-STAT, pAKT, Ras, and NF-κB^17^—we recognized a critical knowledge gap: the precise molecular mechanisms by which nCGA confers its renoprotective effects remain elusive. It remains unclear whether nCGA directly suppresses pyroptosis, an inflammatory form of programmed cell death mediated by the gasdermin-NLRP3 inflammasome axis, which is increasingly implicated in the pathogenesis of DN. In addition, the potential regulatory role of nCGA in modulating specific microRNAs (miRNAs) involved in oxidative stress and inflammasome activation has not been elucidated.

Therefore, the primary aim of this study is to investigate the direct effects of pure nCGA on oxidative stress, inflammatory responses, and pyroptosis in a glucolipotoxicity-induced mesangial cell model with a specific focus on its modulation of inflammasome signaling and miRNA expression. By uncovering these mechanistic pathways, this study seeks to establish nCGA as a promising and mechanistically defined therapeutic candidate for the treatment of diabetic nephropathy.

## 2 Materials and Methods

### 2.1 Protein–Protein Interaction (PPI) Network Construction and Gene Ontology (GO) Enrichment

To elucidate the molecular targets of neochlorogenic acid (nCGA) in diabetic nephropathy, 29 overlapping genes associated with inflammation, DN, and nCGA were identified from the GeneCards, OMIM, TCMSP, and HERB databases. A Venn diagram was constructed using the Bioinformatics platform (https://www.bioinformatics.com.cn/). These genes were subsequently analyzed through the STRING database (version 11.5; confidence score ≥0.4, *Homo sapiens*) to construct a PPI network, which was then visualized in Cytoscape (version 3.10.0). The CytoHubba plugin in Cytoscape was used to identify hub genes based on Maximal Clique Centrality (MCC). The top 10 hub genes were highlighted with color intensity reflecting the degree of connectivity. Among them, NFKB1, TNF, and PTGS2 emerged as the top-ranked nodes, underscoring their importance in inflammatory signaling and as potential nCGA targets.

Furthermore, transcription factor regulatory analysis was performed using Metascape (https://metascape.org/) in combination with the TRRUST database. Pathway enrichment analysis was carried out through STRING coupled with the Reactome database, and the enrichment results were visualized as bubble plots. The statistical strength of enrichment was expressed as log10 (observed/expected), representing the magnitude of enrichment relative to a random network of equal size.

### 2.2 Preparation of nCGA Isomer

nCGA, sourced from Chengdu Alfa Biotechnology CO., Ltd. in Chengdu, China, had a purity of 98%. It was dissolved in dimethyl sulfoxide to create a stock solution at a concentration of 100 mM. This stock solution was then diluted according to the requirements of subsequent experiments.

### 2.3 Chemicals and Reagents

Dulbecco’s modified Eagle’s medium (DMEM), l-glutamine, penicillin–streptomycin mixed antibiotics, fetal bovine serum (FBS), trypsin-EDTA, and phosphate-buffered saline (PBS) were purchased from Gibco/BRL (Gaithersburg, MD). Primary antibodies against β-actin (Invitrogen, MA5-11866), NFκb p65 (Santa Cruz, sc-109), p-NFκb (Abclonal, AP0123), IKB (Santa Cruz, sc-847), p-IKB (Cell Signaling, no. 9246), Nrf2 (Santa Cruz, sc-722), TNF-α (Elabscience, E-AB-40015), MCP-1 (Santa Cruz, sc-28879), ICAM-1 (Santa Cruz, sc-107), COX-2 (Santa Cruz, sc-19999), iNOS (Santa Cruz, sc-7271), NLRP3 (Bioss, bs-10021R), GSDMD (Invitrogen, PA5-104324), IL-1β (abcam, ab283818), and Caspase-1 p20 (Bioss, bs-10442R).

### 2.4 Cell Treatment and Transfection

MES-13 SV40 mouse glomerular mesangial cells (MES-13) were subjected to a high-stress environment using a combination of 25 mM high glucose (HG) and 80 μM oleic acid (OA) to simulate kidney injury. miRNA mimics and inhibitors, along with the corresponding negative control, were acquired from GeneDireX (Las Vegas, NV). The transfection of these components was carried out using T-Pro nonliposomal transfection reagent II (T-Pro NTR II) from T-Pro Biotechnology, based in Taipei, Taiwan. Nucleotide primers (5′–3′) used for reverse transcription were GTTGGCTCTGGTGCAGGGTCCGAGGTATTCGCACCAGAGCCAACTCCTCC for miR-709 and CGCTGCAGTTGGCTCTGGTGCAGGGTCCGAGGTATTCGCACCAGAGCCAA for 30a.

The primers (5′–3′) used for real-time PCR were as follows: miR-709 forward, GGAGGCAGAGGCAGGA; 30a forward, CGATTGGCAGTGTCTTAGCT; and universal reverse primer, GTGCAGGGTCCGAAGT.

### 2.5 Cell Viability Assay

Cell viability was assessed using the MTT assay. Initially, 2 × 10^5^ cells were seeded in a 24-well plate with 10% v/v FBS culture medium and allowed to attach for 24 h. Subsequently, these cells were treated with varying concentrations of nCGA (20–100 μM) for 24 h. Post-treatment, cells were washed twice with PBS and then incubated with MTT reagent (5 mg/mL; Sigma-Aldrich) at 37 °C for 2 h. The supernatant was removed, and 2-propanol was added to solubilize the formazan. The absorbance of the solution was measured at 563 nm using a U-2900 spectrophotometer (Hitachi, Tokyo, Japan). The viability percentage was calculated by comparing these values to those from DMSO-treated control cells, and the results were validated through three independent experiments for statistical reliability.

### 2.6 Animal Experiments

This study was conducted in compliance with the Guide for the Care and Use of Laboratory Animals of the National Institute of Health. The experimental protocol was approved by the Animal Model Experimental Ethics Committee of Chung Shan Medical University (approval no. 2433). All mice used were 6-week-old male BKS.Cg-Dock7m+/+Leprdb/JNarl (db/db) mice, obtained from the National Laboratory Animal Center in Taipei, Taiwan. The mice were housed in a controlled environment (22 ± 2 °C, 65 ± 5% relative humidity, and a 12 h light/dark cycle) with ad libitum access to water and standard chow. The db/db mice served as a model of obesity to replicate symptoms of type II diabetes mellitus in humans, induced through genetic mutations in leptin receptors, leading to hyperphagia, obesity, insulin resistance, and ultimately type II diabetes mellitus. This animal model has already been published^17^ and is well-established in the study of diabetes mellitus. The db/db mice were divided into two groups (*n* = 6 per group): the control group (db/db mice with a normal diet) and the HFD group (db/db mice with a high-fat diet).

### 2.7 miRNA Isolation

Total RNA was isolated from MES-13 cells, transcribed into cDNA, and analyzed via a stem-loop reverse transcription-polymerase chain reaction (RT-PCR). Real-time PCR was conducted by using the LightCycler 480 SYBR Green I Master mix and the LightCycler 480 real-time PCR machine (Roche Applied Science). The relative expression levels of the miRNA were normalized to those of the internal invariant control RNU6B (6B). Each assay was carried out in triplicate, and the data were analyzed by using the 2-ΔCt method.

### 2.8 Western Blotting Analysis

MES-13 SV40 mouse glomerular mesangial cells were lysed using RIPA buffer following a 24 h treatment with various reagents. After treatment and lysis, the cell lysates were centrifuged at 12,000*g* at 4 °C for 25 min to separate the supernatant. Protein samples, each weighing 50 μg, were processed through sodium dodecyl sulfate-polyacrylamide gel electrophoresis and then transferred onto nitrocellulose membranes (Millipore, Bedford, MA). The membranes were blocked using a solution of 5% nonfat milk powder and 0.1% Tween 20 in Tris-buffered saline (TBS), followed by overnight incubation at 4 °C with the specified primary antibody: β-actin (1:5000), NFκb p65 (1:1000), p-NFκb (1:1000), IKB (1:1000), p-IKB (1:500), Nrf2 (1:1000), TNF-α (1:1000), MCP-1 (1:500), ICAM-1 (1:1000), COX-2 (1:1000), iNOS (1:1000), NLRP3 (1:1000), GSDMD (1:1000), IL-1β (1:1000), and Caspase-1 p20 (1:750). Subsequently, the membranes were washed three times with 0.1% Tween 20 in TBS and incubated with a horseradish-peroxidase-conjugated secondary antibody (GE Healthcare, Little Chalfont, Buckinghamshire, UK). Proteins were visualized using enhanced chemiluminescence (ECL) and detected on an ECL hyperfilm using a Fujifilm LAS-4000 system (Tokyo, Japan). Protein quantification was performed through densitometry using Fujifilm Multi Gauge V2.2 software (Tokyo, Japan).

### 2.9 Determination of Reactive Oxygen Species

Intracellular reactive oxygen species (ROS) were quantified using 2,7-dichlorodihydrofluorescein diacetate (H2DCFDA), a nonfluorescent probe that reacts with ROS to form a fluorescent compound. The process involves the enzymatic conversion of H2DCFDA by viable cells into 2,7-dichlorodihydrofluorescein (HDCF), which subsequently reacts with intracellular ROS to form 2,7-dichlorofluorescein (DCF). This fluorescent molecule remains trapped within the cells, allowing for quantification of the ROS levels. The assay was applied to nCGA-treated MES cells, which were incubated with 10 μM H2DCFDA for 30 min following nCGA exposure (20 ng/mL). Fluorescence was measured using a Becton-Dickinson FACS-Calibur flow cytometer, and the data, expressed as mean ± SE from three independent experiments, were normalized to control cells with their fluorescence arbitrarily set as 1.

### 2.10 Statistical Analysis

All statistical analyses were performed using GraphPad Prism (ver. 8.0.2, GraphPad Software, San Diego, CA). For comparisons involving multiple groups, a one-way analysis of variance (ANOVA) was conducted, followed by the Bonferroni post-hoc test to account for multiple comparisons. All experiments were performed in triplicate, and data were presented as mean ± standard deviation (SD) unless otherwise specified.^21^

## 3 Results

### 3.1 Natural Abundance of Neochlorogenic Acid (nCGA)

Figure 1 provides a quantitative summary adapted from previous review articles, illustrating the wide variability in neochlorogenic acid (nCGA) concentrations across various plant-based food sources^22–28^ (Figure 1). Highbush blueberries exhibited the highest mean nCGA content, with concentrations ranging from 37 to 76 g·kg^–1^ of dry weight (dw). Among plant products, green coffee and roasted coffee also contained notably high levels of nCGA, ranging from 29.9 to 65.5 and 27.9 to 52.0 g·kg^–1^ dw, respectively. In addition, other sources such as sunflower seeds, mulberry leaves, and eggplant indicated moderate to high nCGA concentrations, indicating their potential as significant dietary sources of this polyphenolic compound.

**Figure 1:**
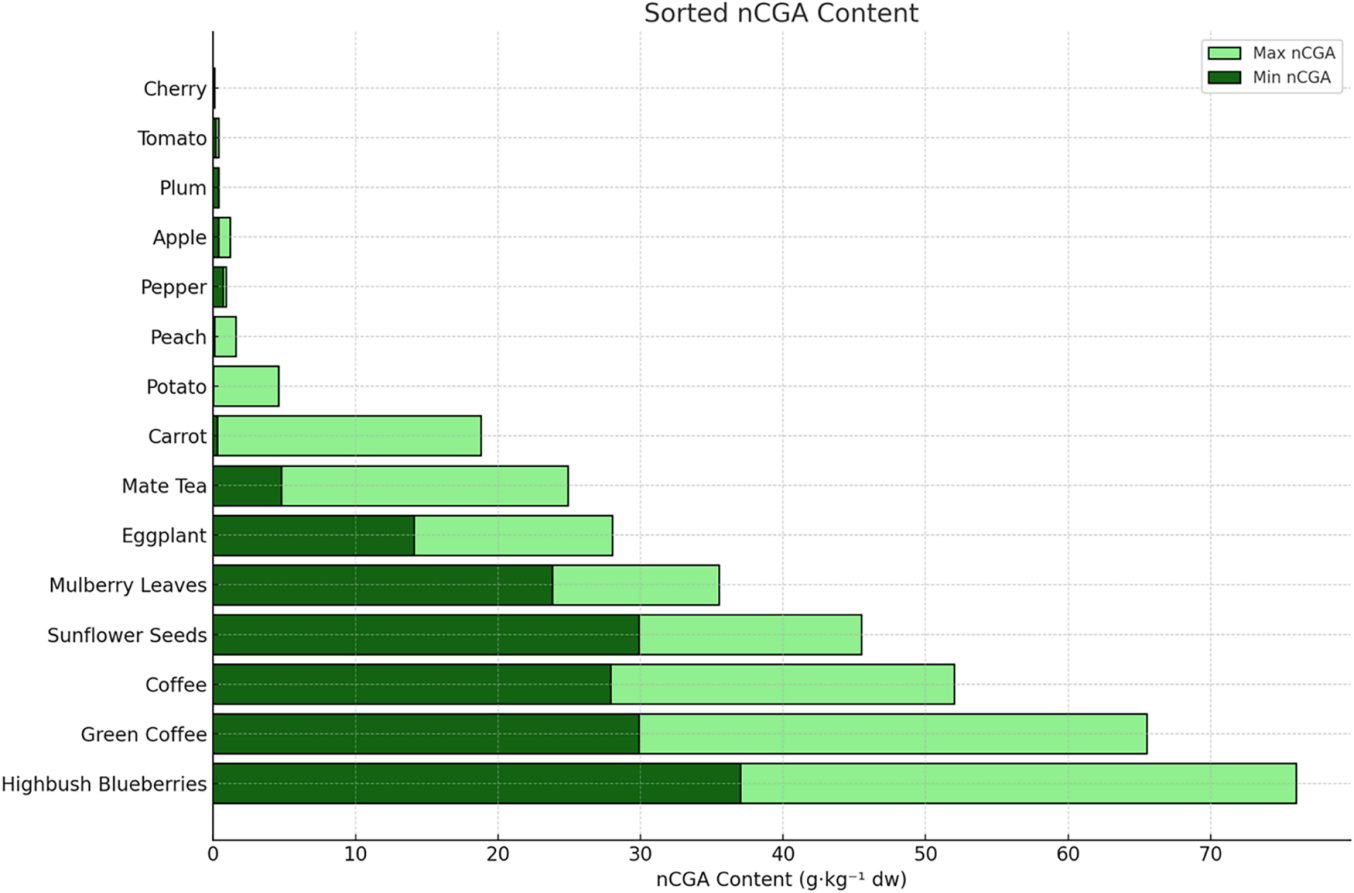
Comparative Distribution of Neochlorogenic Acid (nCGA) in Selected Vegetables, Fruits, and Plant Products. Bar chart representing the minimum and maximum concentrations of neochlorogenic acid (nCGA; 5-*O*-caffeoylquinic acid) in various vegetables, fruits, and plant-derived products, expressed as g·kg^–1^ dry weight (dw). Data are sorted by average nCGA content in descending order. Dark green bars indicate minimum concentrations, while light green bars represent maximum concentrations.

### 3.2 Identification of Common Targets among Inflammation, Diabetic Nephropathy, and nCGA

To elucidate the potential mechanisms of neochlorogenic acid (nCGA) in diabetic nephropathy, we identified 29 overlapping genes associated with inflammation, diabetic nephropathy, and nCGA targets through the integrative analysis of GeneCards, OMIM, TCMSP, and HERB databases (Figure 2A). These overlapping genes were used to construct a protein–protein interaction (PPI) network via the STRING database (Figure 2B), which revealed a complex and densely interconnected network, indicating a strong biological relevance and coordinated regulation among these genes.

**Figure 2:**
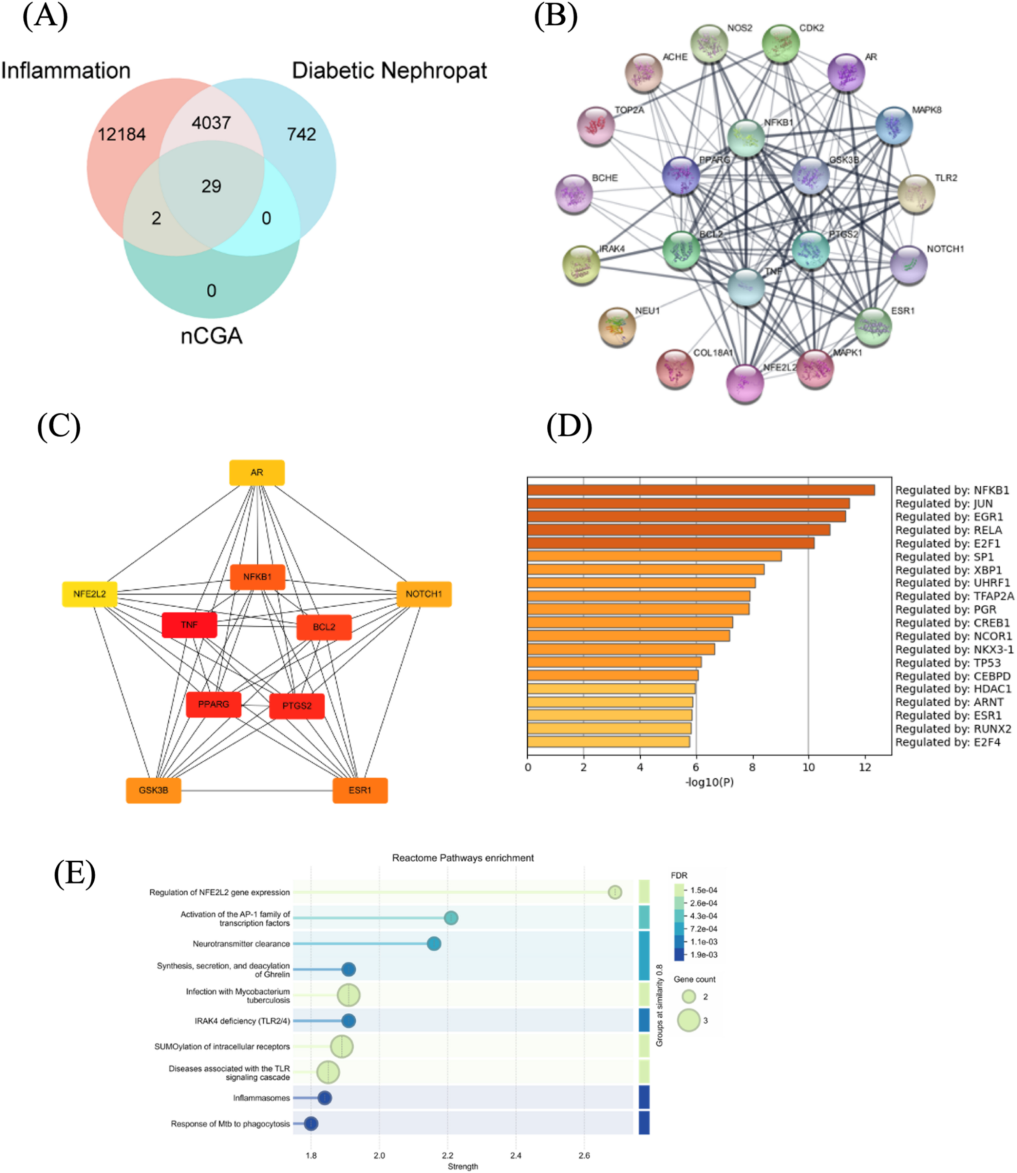
PPI Network and Regulatory Analysis of Inflammation-, Diabetic Nephropathy-, and nCGA-Related Targets. (A) Venn diagram showing shared genes among inflammation, diabetic nephropathy, and nCGA targets from GeneCards, OMIM, TCMSP, and HERB. (B) PPI network constructed via STRING; nodes represent genes, and edge thickness indicates interaction strength. (C) Top 10 hub genes identified using CytoHubba; (red color indicates top order-highly interactive; orange color indicates -moderate interactive; and yellow color indicates -mild interactive). (D) Transcription factors regulating key genes identified using TRRUST; bars represent -log10 (*p*-value). (E) Reactome pathway enrichment of overlapping genes, showing key pathways in inflammation, immunity, and transcription. Dot size indicates gene count; color reflects FDR significance.

Using the CytoHubba plugin in Cytoscape, the top 10 hub genes were identified based on their degree of connectivity within the PPI network (Figure 2C). Notably, NFKB1, TNF, and PTGS2 (COX-2) emerged as central nodes with high interaction scores, suggesting their pivotal role in mediating the inflammatory response and contributing to the pathogenesis of diabetic nephropathy. These genes are known to regulate proinflammatory cytokine signaling, oxidative stress, and renal injury, further supporting their biological significance. Their prominent positioning in the network highlights them as potential molecular targets for nCGA intervention and therapeutic modulation.

Transcription factor analysis using the TRRUST database revealed NFKB1 as the most significantly enriched upstream regulator of the overlapping genes (Figure 2D). As a central component of the NF-κB signaling pathway, NFKB1 plays a pivotal role in mediating inflammatory responses, oxidative stress, and immune activation—all of which are key contributors to the development of diabetic nephropathy.

Consistently, pathway enrichment analysis using the Reactome database indicated that several of the shared targets are involved in NF-κB-related pathways, including regulation of NF-κB gene expression, activation of TLR signaling, and inflammasome responses (Figure 2E). These findings highlight the NF-κB axis as a major signaling hub, potentially modulated by nCGA, in the context of an inflammation-driven renal injury.

### 3.3 Cytotoxicity Assessment in MES-13 Cells

We conducted cytotoxicity assays at various doses of oleic acid (OA). We discovered that doses ranging from 80 to 160 μM resulted in measurable damage to MES-13 cells (Figure 3A), with an estimated lethal dose of approximately 135 μM. Given the potential influence of drug-induced cellular damage on the induction effects, we selected 80 μM as the induction dose for OA. Subsequently, we examined the effects of different concentrations of nCGA on cell viability. We observed that nCGA did not significantly affect cell viability at concentrations ranging from 50 to 200 μM (Figure 3B). In addition, we explored the effects of nCGA on glucolipotoxicity-induced MES-13 cells, and we noticed that nCGA did not compromise cell viability at either concentration tested (100 and 200 μM). Taken together, these findings suggest that nCGA influences cellular function instead of inducing cellular death (Figure 3C).

**Figure 3:**
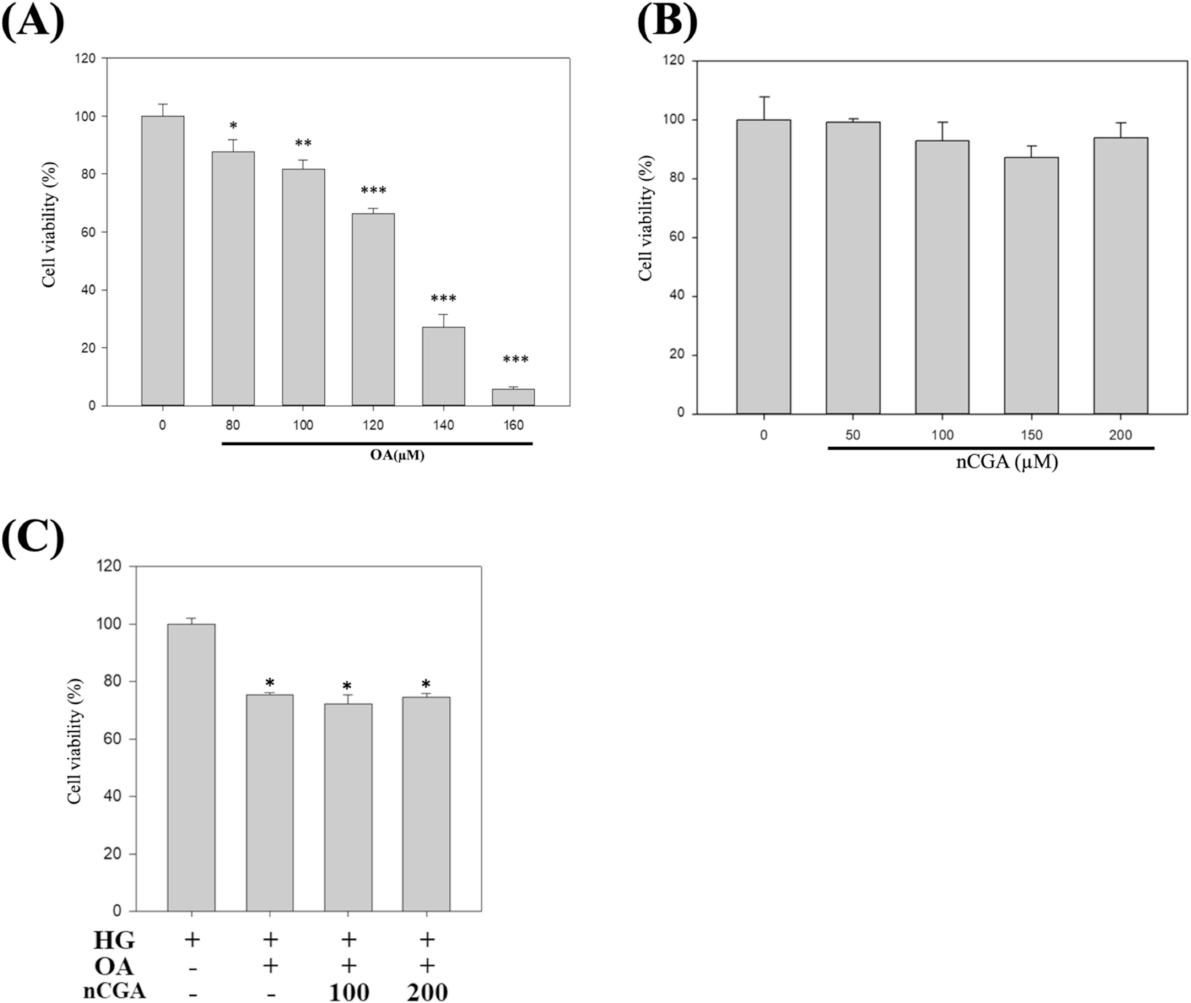
Effect of nCGA and OA in MES-13 mesangial cells. MES-13 mouse mesangial cells were treated with the indicated concentration of oleic acid (A) and nCGA (B) for 24 hr. (C) MES-13 mouse mesangial cells were treated with high glucose (25 mM) and oleic acid and nCGA (100 and 200 μM) for 24 hr. Quantification of the cell viability. Data are shown as mean ± SD. Results were statistically analyzed with the Student *t-*test. *p < 0.05 compared to HG group, ** *p* < 0.01 compared to HG group, and ### *p* < 0.001 compared to HG group.

### 3.4 nCGA Enhances Nrf2 Signaling and Reduces the Activity of the NF-κB Family, Thereby Downregulating the Levels of Inflammatory Factors

To determine the protective effects of nCGA on MES-13 cells under glucolipotoxic conditions, we examined the expression levels of Nrf2 and NF-κB signaling pathways, which play pivotal roles in cellular defenses against oxidative damage and inflammation. We discovered that treatment with nCGA substantially promoted the activation of Nrf2, which, in turn, enhanced the cellular antioxidant response. These findings were confirmed by the upregulation of the expression of various antioxidant genes downstream of Nrf2, which mitigate oxidative stress within cells (Figure 4A).

**Figure 4:**
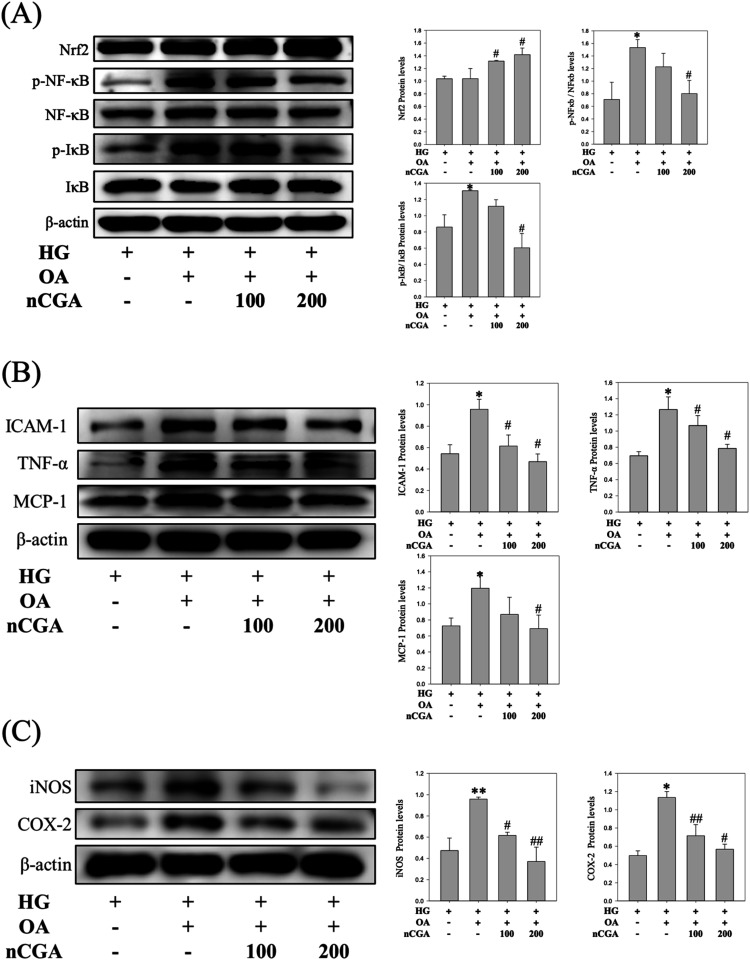
nCGA’s effects on glucolipotoxicity-induced MES-13 mesangial cells, where it promoted Nrf2 expression, inhibited NF-κB phosphorylation, and decreased the protein expression of oxidative stress-associated inflammatory factors. (A) MES-13 cells were subjected to Western blotting to analyze Nrf2 and NF-κB expression and quantification of the protein expression. (B) MES-13 cells were subjected to Western blotting to analyze ICAM-1, TNF-α, and MCP-1 expression and quantification of the protein expression. (C) MES-13 cells were subjected to Western blotting to analyze COX-2 and iNOS expression and quantification of the protein expression. Data are shown as mean ± SD. Results were statistically analyzed using ANOVA, followed by the Bonferroni post-hoc test. A *p*-value (*) of <0.05 was considered statistically significant compared to the HG + OA group.

Under nonactivated conditions, NF-κB dimers are typically bound to IκB (inhibitor of kappa B) in the cytosol; thus, they remain inactive. Exposure to glucolipotoxicity (HG + OA) conditions activates NF-κB, which, in turn, causes the phosphorylation and degradation of IκB through the ubiquitin–proteasome system. This process liberates NF-κB, which subsequently enters the nucleus and induces the production of proinflammatory cytokines. Induction of glucolipotoxicity (HG + OA) significantly increases the phosphorylation levels of proteins involved in the NF-κB pathway, including NF-κB and IκB. In this study, treatment with nCGA (HG + OA + nCGA) resulted in the marked reduction of these phosphorylated proteins, particularly in p-NF-κB and p-IκB, indicating that nCGA effectively inhibited inflammatory responses (Figure 4A).

In our study, the analysis of the inflammatory factors downstream of the NF-κB pathway revealed the upregulation of the levels of inflammatory proteins such as TNF-α, MCP-1, and ICAM-1 in the glucolipotoxicity (HG + OA) group. In the nCGA (HG + OA + nCGA) group, we observed that the levels of these inflammatory markers were significantly lower, and the extent of reduction was positively correlated with the dose of nCGA, than those of the glucolipotoxicity (HG + OA) group (Figure 4B). Taken together, these findings indicate that nCGA effectively mitigates the inflammatory response induced by glucolipotoxicity, as confirmed by the significant reduction in key inflammatory markers; the effects were enhanced at higher doses of nCGA.

In addition to exploring inflammatory pathways, we evaluated the levels of inflammatory proteins such as COX-2 and iNOS, influenced by both inflammatory responses and oxidative stress. Our results indicated that the induction of glucolipotoxicity (HG + OA group) upregulated the levels of these inflammatory proteins, which were significantly higher than those observed in the HG group. After treatment with nCGA (HG + OA + nCGA group), we observed a significant reduction in the expression of these activated inflammatory proteins, and the degree of reduction was positively correlated with the dose of nCGA (Figure 4C).

### 3.5 nCGA Mitigates Pyroptosis and Inflammation in Glucolipotoxicity-Induced MES-13 Cells

To investigate the protective effects of nCGA on pyroptosis and inflammation in MES-13 cells under glucolipotoxic conditions, we examined the expression levels of key pyroptosis-related proteins. Our results revealed that exposure to glucolipotoxic conditions (HG + OA) significantly upregulated the expression levels of NLRP3, GSDMD, GSDMD-N, IL-1β, and caspase-1, indicating the activation of pyroptotic pathways and an increase in inflammatory responses. In particular, the presence of the cleaved N-terminal fragment GSDMD-N, a hallmark executioner of pyroptosis, further confirmed pyroptotic activation under glucolipotoxic stress.^29^ The results clearly show that GSDMD-N expression was markedly increased under glucolipotoxic conditions (HG + OA), and treatment with nCGA significantly reduced its level in a dose-dependent manner. Treatment with nCGA (HG + OA + nCGA) markedly reduced the expression levels of these proteins, including GSDMD-N, in a dose-dependent manner, especially at a higher concentration of 200 μM. These findings suggest that nCGA effectively inhibits the activation of the NLRP3 inflammasome and its downstream pyroptotic mediators (Figure 5A,B). Moreover, to further confirm the occurrence of pyroptotic cell death, Annexin-V/PI flow cytometry analysis was performed. Exposure to HG + OA markedly increased Annexin-V positivity, reflecting enhanced phosphatidylserine externalization and membrane disruption, which are characteristic of both apoptosis and pyroptosis (Figure 5C). These findings were consistent with Western blot data showing upregulation of NLRP3, GSDMD, IL-1β, and Caspase-1, indicating activation of the pyroptotic pathway. Importantly, treatment with nCGA significantly reduced Annexin-V fluorescence in a dose-dependent manner, with 200 μM nearly restoring the levels observed in the HG group. These results suggest that nCGA mitigates glucolipotoxicity-induced pyroptosis and preserves the cell membrane integrity. To further investigate the cellular localization and expression of pyroptosis-related proteins, we performed immunofluorescence staining for NLRP3, caspase-1, and GSDMD in MES-13 cells. As shown in Figure 5D–F, exposure to glucolipotoxic conditions (HG + OA) markedly enhanced the fluorescence intensity of NLRP3, caspase-1, and GSDMD compared with the HG group, indicating robust activation of the pyroptotic pathway. By contrast, treatment with nCGA reduced the fluorescence signal of these proteins in a dose-dependent manner, particularly at 200 μM, where the staining intensity was substantially diminished. These results further confirm that nCGA suppresses the activation and cellular localization of key pyroptosis mediators in glucolipotoxicity-induced renal mesangial cells.

**Figure 5:**
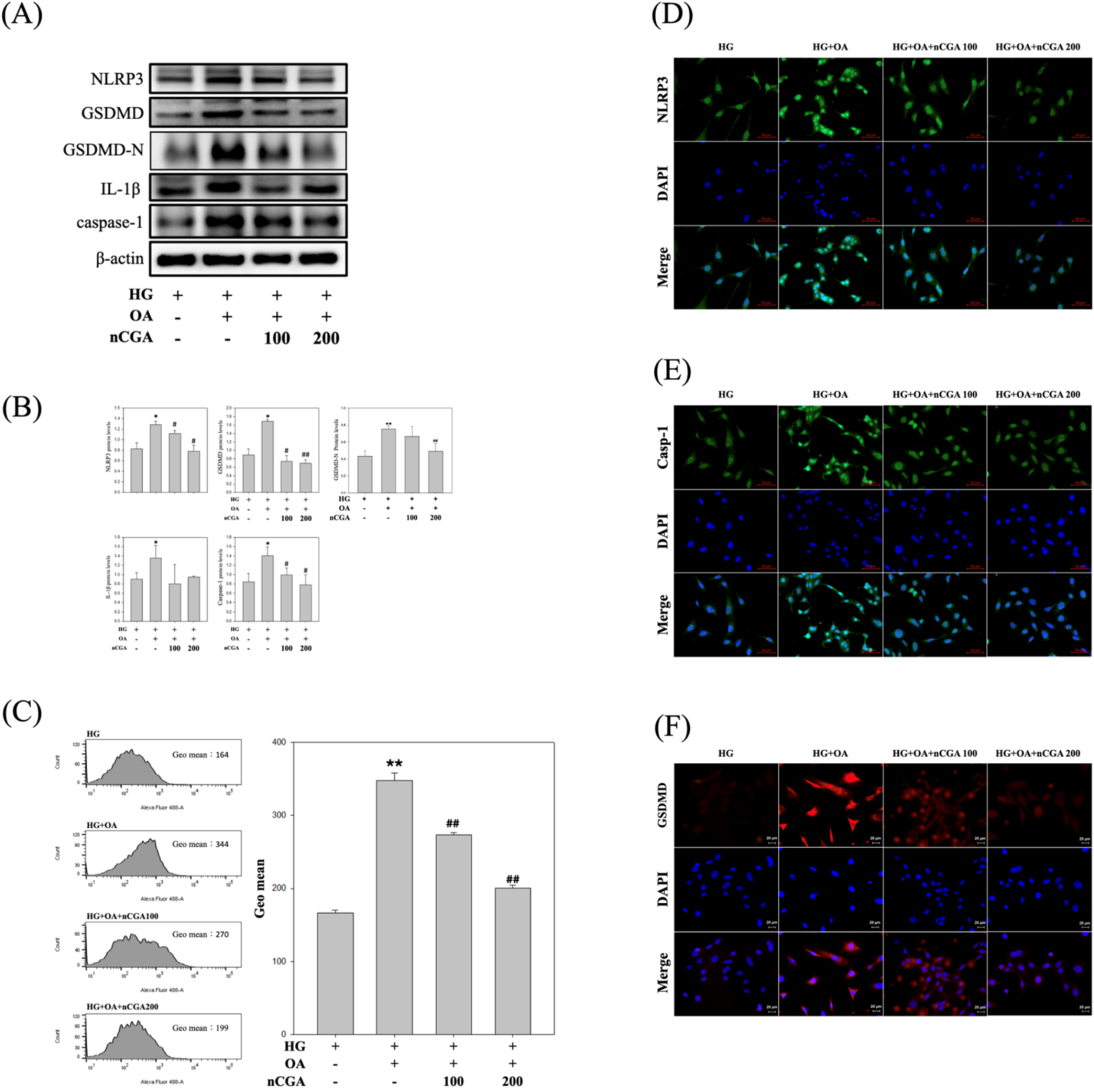
Effect of nCGA in decreasing both the pyroptosis and inflammation levels in glucolipotoxicity-induced MES-13 cells. (A) MES-13 cells were subjected to Western blotting to analyze NLRP3, GSDMD, IL-1β, and Caspase-1 expression. (B) Quantification of the protein expression. (C) Annexin-V/PI flow cytometry analysis was performed to detect phosphatidylserine externalization, a marker of both apoptosis and pyroptosis-associated membrane disruption. (D–F) Immunofluorescence staining for the expression and cellular localization of pyroptosis markers. Data are shown as mean ± SD. Results were statistically analyzed using ANOVA, followed by the Bonferroni post-hoc test. *p < 0.05 was considered statistically significant compared to the HG + OA group.

### 3.6 nCGA Mitigates the Level of Reactive Oxygen Stress and Mitochondrial Membrane Potential in Glucolipotoxicity-Induced MES-13 Cells

In this study, we measured the levels of ROS in MES-13 cells.^30^ To determine the effect of nCGA on oxidative stress in cells under glucolipotoxicity conditions, we used 2′,7′-dichlorodihydrofluorescein diacetate (DCFDA) staining and flow cytometry. Our results indicated that the levels of ROS were significantly higher in the induced group (HG + OA) than in the HG group, suggesting that glucolipotoxicity increased the levels of ROS, which in turn increased the degree of oxidative stress in the cells. By contrast, we observed that the levels of ROS were significantly lower in the HG group than in the induced group (HG + OA), and they decreased with increasing doses of nCGA. Taken together, these findings indicate that nCGA can mitigate the degree of oxidative stress in kidney cells under glucolipotoxicity conditions (Figure 6A).

**Figure 6:**
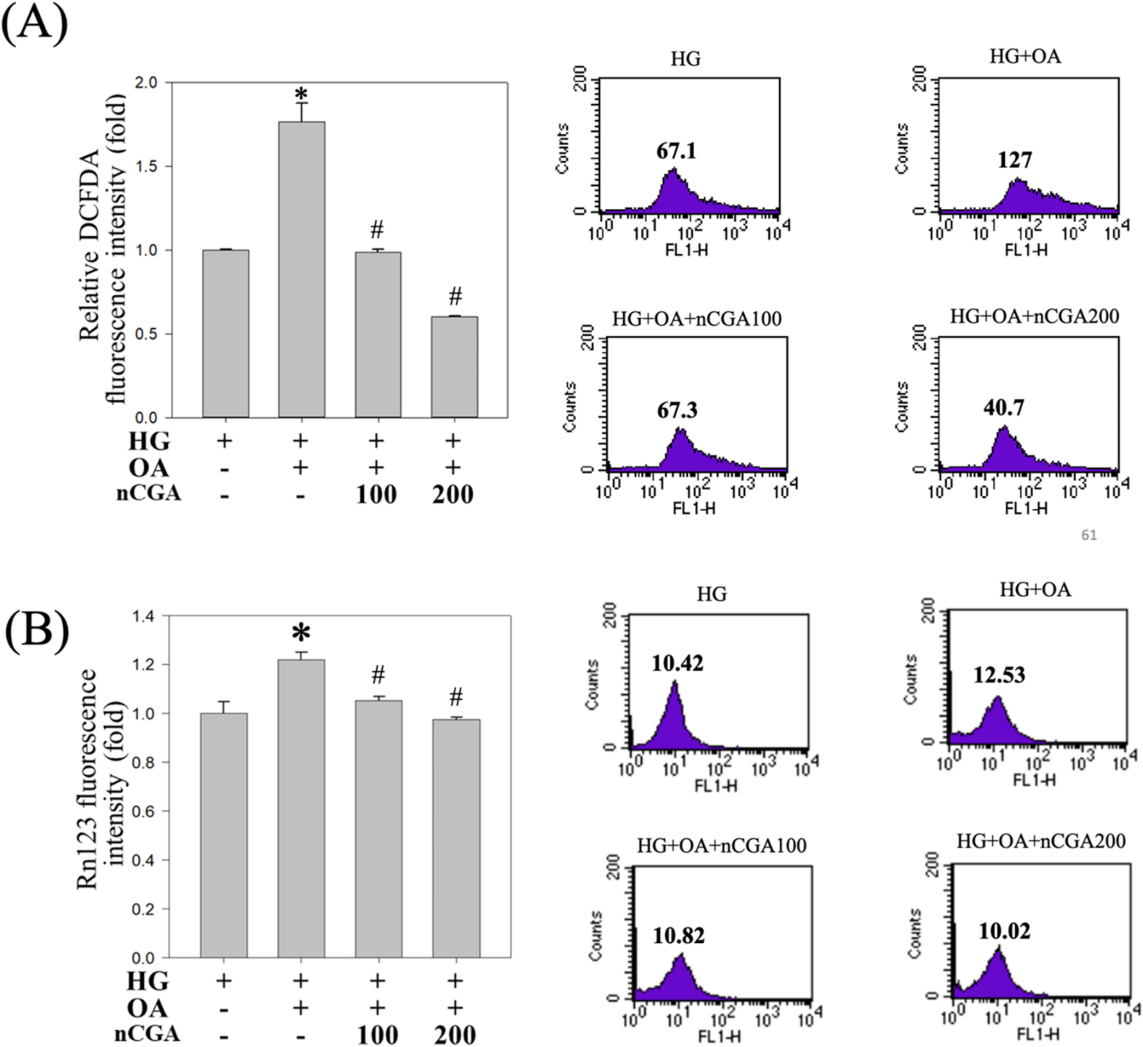
Effect of nCGA in decreasing both the reactive oxygen stress level and the mitochondrial membrane potential level in glucolipotoxicity-induced MES-13 cells. (A) MES-13 mouse mesangial cells were treated with high glucose (25 mM) and oleic acid (80 μM) and nCGA (100 and 200 μM) for 24 hr. Determination of cellular ROS by the DCFDA assay, and quantification of the ROS level by flow cytometry. (B) MES-13 mouse mesangial cells were treated under the same conditions. Determination of cellular ROS by the Rhodamine123 assay, and quantification of the MMT level by flow cytometry. Data are shown as mean ± SD. Results were statistically analyzed using ANOVA, followed by the Bonferroni post-hoc test. *p < 0.05 compared to the HG group, # *p* < 0.05 compared to the HG + OA group.

To identify the mechanism of oxidative stress induced by glucolipotoxicity, we used dihydrorhodamine 123 (DHR123) with flow cytometry for mitochondrial cell staining. This fluorescent molecule accumulates in response to mitochondrial activity, serving as an indicator of the mitochondrial membrane potential and function. In this study, we utilized the fluorescence properties of DHR123 for evaluating the degree of oxidative stress caused by glucolipotoxicity. Our results indicated that the intensity of fluorescence was significantly higher in the glucolipotoxicity-induced group than in the HG group (Figure 6B). In addition, we observed that nCGA inhibited changes in mitochondrial membrane potential with a pattern similar to that observed with DCFDA (Figure 6A). Taken together, these findings suggest a strong connection between glucolipotoxicity-induced oxidative stress and mitochondrial function, further confirming the protective effects of nCGA.

### 3.7 Effect of nCGA on the Expression of miRNAs in Diabetic Mice on HFDs

In our previous study involving an animal model, the study examined whether nCGA ameliorates glucolipotoxicity-induced diabetic nephropathy (DN) in db/db mice fed a high-fat diet (HFD).^17^ Their findings indicated that both mulberry leaf extract and its active compound, neochlorogenic acid (nCGA), showed significant potential in managing glucolipotoxicity-induced DN by targeting lipid metabolism and key molecular pathways. Building on these findings, the present study aimed to further elucidate the molecular mechanisms of nCGA, particularly through its regulation of miRNAs. To this end, we employed miRNA microarray validation (Mouse & Rat miRNA OneArray; Phalanx Biotech Group, Hsinchu, Taiwan) and quantitative reverse transcription-polymerase chain reaction (qRT-PCR) analysis to compare the expression of miRNAs in db/db mice fed an HFD versus those on a standard diet. Comparing HFD-fed and standard diet-fed db/db mice allows us to specifically assess how dietary lipotoxic stress modulates miRNA expression in the diabetic state, providing insight into miRNA-related mechanisms underlying DN progression.

As shown in Figure 7A, among the top 30 downregulated miRNAs identified through our microarray analysis, miR-29b-3p, miR-26a-5p, and miR-30a-3p ranked among the most significantly suppressed in HFD-fed db/db mice compared to the controls. However, based on previous literature, only miR-29b and miR-30a have been functionally implicated in the regulation of the NF-κB signaling pathway, which plays a critical role in inflammation and diabetic nephropathy.^31^ Therefore, we selected miR-29b and miR-30a for subsequent validation and mechanistic studies. As shown in Figure 7B, glucolipotoxic stress (high glucose and oleic acid) significantly reduced the expression levels of both miR-29b and miR-30a in MES-13 mesangial cells. Treatment with nCGA (100 and 200 μM) resulted in a modest restoration of miR-29b levels, while miR-30a expression was markedly upregulated in a dose-dependent manner. These findings suggest that miR-30a may serve as a primary miRNA mediator through which nCGA attenuates inflammation via the suppression of NF-κB activity.

**Figure 7:**
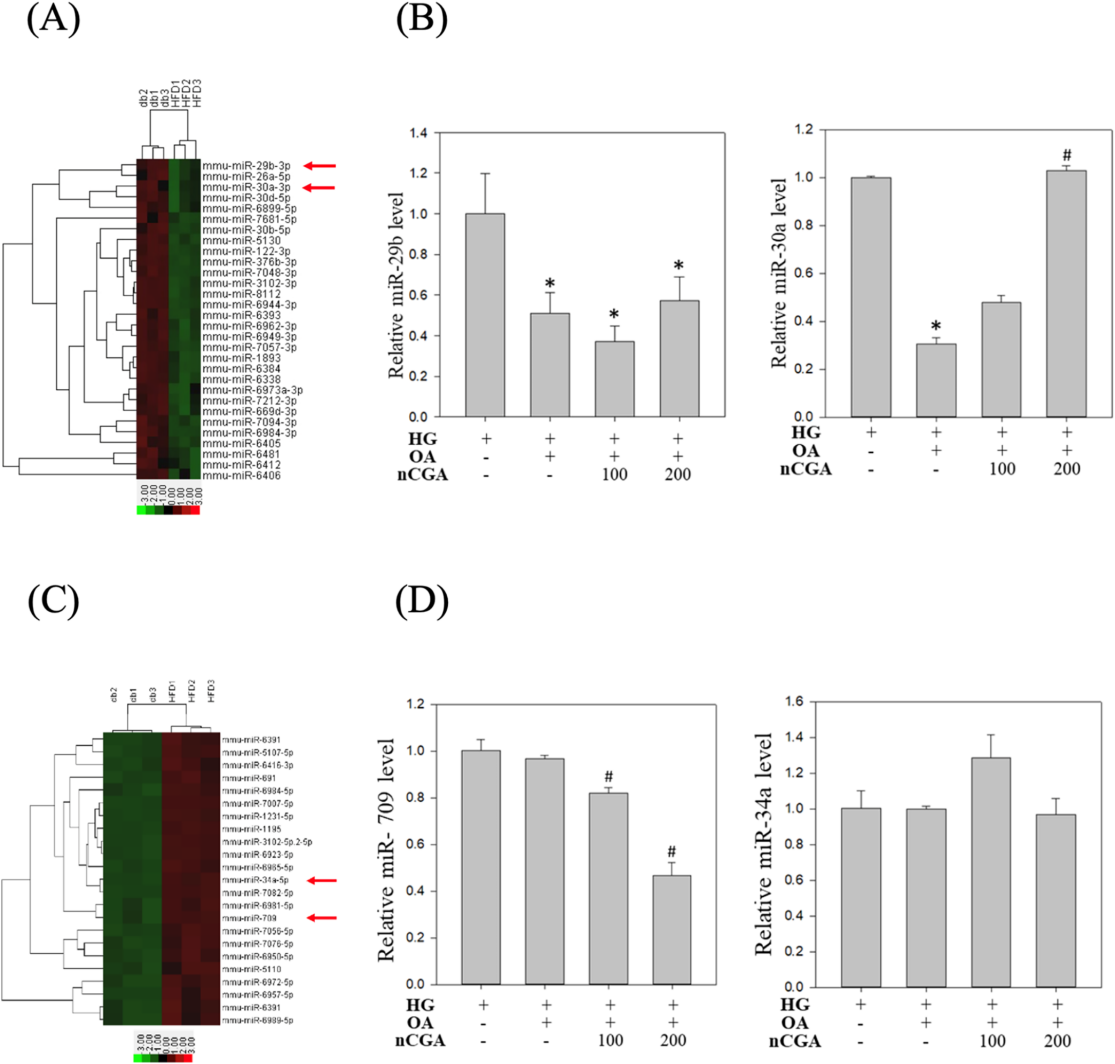
Mechanism of nCGA’s influence on renal pathology through miRNA. (A) A subset of differential genes was selected for clustering analysis. Representation of the top 30 downregulated genes in red and green colors. miRNAs were detected both by microarray analysis between HFD-fed db/db mice and normal diet-fed db/db mice. (B) MES-13 cells were subjected to real-time PCR to analyze miR-29b and miR-30a expression. (C) A subset of differential genes was selected for clustering analysis. Representation of the top 23 upregulated genes in red and green colors. miRNAs were detected both by microarray analysis between HFD-fed db/db mice and normal diet-fed db/db mice. (D) MES-13 cells were subjected to real-time PCR to analyze miR-709 and miR-34a expression. Results were statistically analyzed using ANOVA, followed by the Bonferroni post-hoc test. *p < 0.05 compared to the HG group and # *p* < 0.05 compared to the HG + OA group.

On the other hand, among the 23 upregulated miRNAs identified from our microarray analysis in the kidneys of HFD-fed mice (Figure 7C), miR-709 and miR-34a drew particular attention due to their previously reported involvement in the regulation of Nrf2 signaling.^32^ miR-709 was significantly elevated under glucolipotoxic conditions and was dose-dependently suppressed by nCGA treatment, although miR-34a levels did not significantly change with nCGA treatment (Figure 7D). Therefore, miR-709 was selected as a primary miRNA candidate through which nCGA may attenuate oxidative stress and inflammation, potentially via the suppression of Nrf2 activity.

### 3.8 miR-30a Reduces Inflammation by Inhibiting the NF-κB and TNF-α Signaling Pathways

In this study, we discovered that miR-30a played a role in downregulating the levels of inflammation-related proteins in DN. We used miR-30a inhibitors and mimics to determine whether miR-30a exerts its effects through the NF-κB and TNF-α signaling pathways. Our results indicated that treatment with OH significantly upregulated the levels of NF-κB, TNF-α, and MCP-1 (Figure 8A). However, treatment with nCGA mitigated these effects. By contrast, utilizing an miR-30a inhibitor upregulated the levels of NF-κB, TNF-α, and MCP-1, indicating its involvement in the inflammatory pathway. Further analysis revealed that the introduction of a miR-30a mimic increased the levels of miR-30a (Figure 8B), which in turn suppressed the NF-κB and TNF-α signaling pathways. These results are consistent with the effects observed with the nCGA treatment. Taken together, our findings suggest that miR-30a serves as a key miRNA that downregulates inflammatory responses through the NF-κB and TNF-α pathways, with nCGA acting as a promising compound to prevent DN through the regulation of the NF-κB and TNF-α pathways.

**Figure 8:**
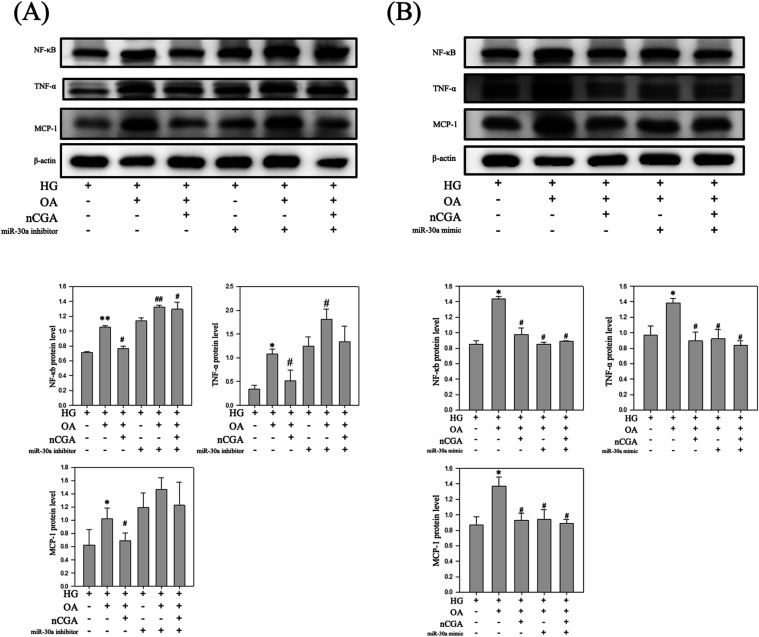
Suppression and induction of miR-30a expression in high glucose, oleic acid, and nCGA conditions. (A) Suppression of miR-30a expression in Western blotting analysis of NF-κB, TNF-α, and MCP-1, and quantification of the protein expression. (B) Suppression of miR-30a induced in Western blotting analysis of NF-κB, TNF-α, and MCP-1, and quantification of the protein expression. Results were statistically analyzed using ANOVA, followed by the Bonferroni post-hoc test. *p < 0.05 compared to the HG group and # *p* < 0.05 compared to the HG + OA group.

### 3.9 miR-709 Increases the Production of ROS by Inhibiting the Nrf2 Signaling Pathway and Upregulates the Levels of Inflammation-Related Proteins

Generally, miR-709 plays a pivotal role in DN by upregulating the levels of ROS through the inhibition of the Nrf2 signaling pathway and the upregulation of the levels of inflammation-related proteins.^16^ Mechanistically, inhibiting miR-709 may hinder the progression of DN by reducing the accumulation of ROS. In this study, treatment with OH significantly downregulated the levels of Nrf2, but treatment with nCGA reversed this effect (Figure 9A). By contrast, treatment with a miR-709 inhibitor upregulated the levels of Nrf2, indicating its involvement in the antioxidative stress pathway. In addition, treatment with OH markedly increased the expression levels of NF-κB, TNF-α, and MCP-1, but treatment with nCGA mitigated these increases. Inhibition of miR-709 further suppressed activation of the NF-κB, TNF-α, and MCP-1 signaling pathways, which are crucial for inflammation reduction.

**Figure 9:**
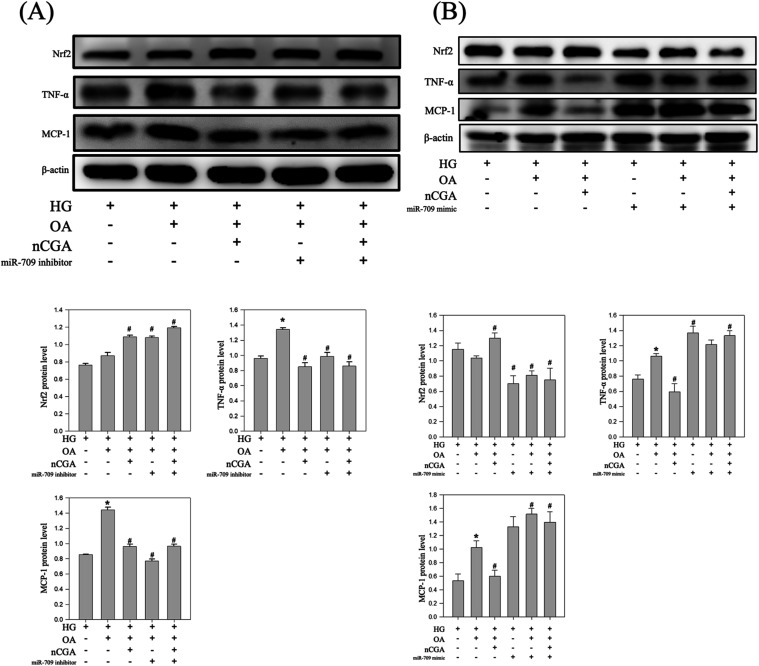
Suppression and induction of miR-709 expression in high glucose, oleic acid, and nCGA conditions. (A) Suppression of miR-709 expression in Western blotting analysis of NF-κB, TNF-α, and MCP-1, and quantification of the protein expression. (B) Suppression of miR-709 induced in Western blotting analysis of NF-κB, TNF-α, and MCP-1, and quantification of the protein expression. Results were statistically analyzed using ANOVA, followed by the Bonferroni post-hoc test. *p < 0.05 compared to the HG group and # *p* < 0.05 compared to the HG + OA group.

We used a miR-709 mimic to further elucidate the role of miR-709 (Figure 9B). Our results indicated that treatment with OH significantly downregulated the levels of Nrf2, but treatment with nCGA counteracted this reduction. By contrast, the coadministration of an miR-709 mimic and nCGA continued to suppress the expression of Nrf2, indicating its importance in the antioxidative stress pathway. These findings indicate that miR-709 is a key regulatory miRNA that modulates the Nrf2 and NF-κB pathways, with nCGA showing potential as a preventive agent against DN.

### 3.10 Regulatory Role of miR-709 and miR-30a in nCGA-Mediated Pyroptosis Suppression

To further examine the involvement of miRNAs in pyroptosis, we performed gain- and loss-of-function assays using miR-709 and miR-30a modulators. Inhibition of miR-709 or overexpression of miR-30a significantly reduced the expression of pyroptosis-related proteins, including NLRP3, GSDMD-N, IL-1β, and caspase-1, under glucolipotoxic conditions (HG + OA). Importantly, nCGA treatment synergistically enhanced these suppressive effects, indicating that nCGA protects mesangial cells partly through miR-709 downregulation and miR-30a upregulation. By contrast, restoration of miR-709 with its mimic or inhibition of miR-30a abolished the protective effect of nCGA, leading to the reactivation of pyroptotic signaling (Figures 10 and 11). These findings confirm that the nCGA-mediated suppression of pyroptosis is closely associated with miRNA regulation.

**Figure 10:**
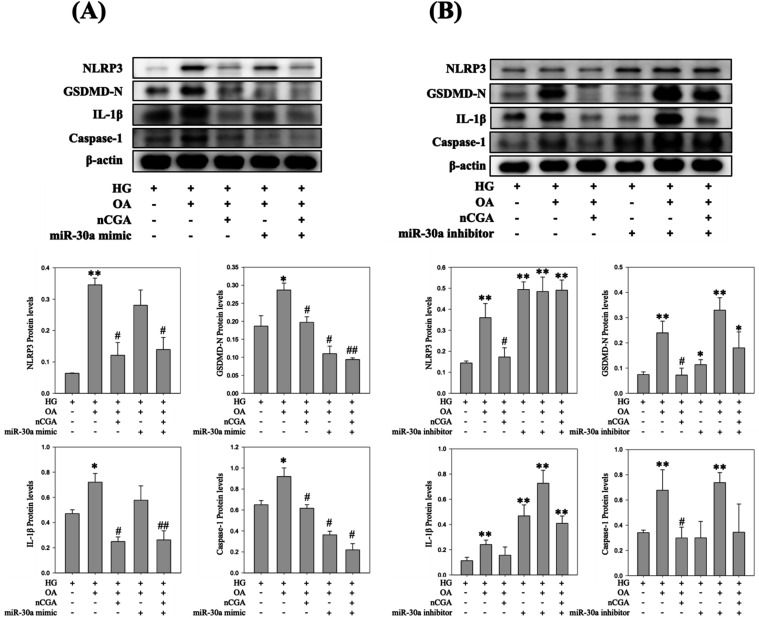
Effect of miR-30a overexpression and miR-30a inhibition on pyroptosis in glucolipotoxicity-induced MES-13 cells. (A) Western blot and quantitative analysis of NLRP3, GSDMD-N, IL-1β, and caspase-1 after treatment with HG, OA, nCGA, and miR-30a mimic. (B) Western blot and quantitative analysis of the same proteins after treatment with the HG, OA, nCGA, and miR-30a inhibitor. Data are shown as mean ± SD *p < 0.05 compared to the HG group and #*p* < 0.05 compared to the HG + OA group.

**Figure 11:**
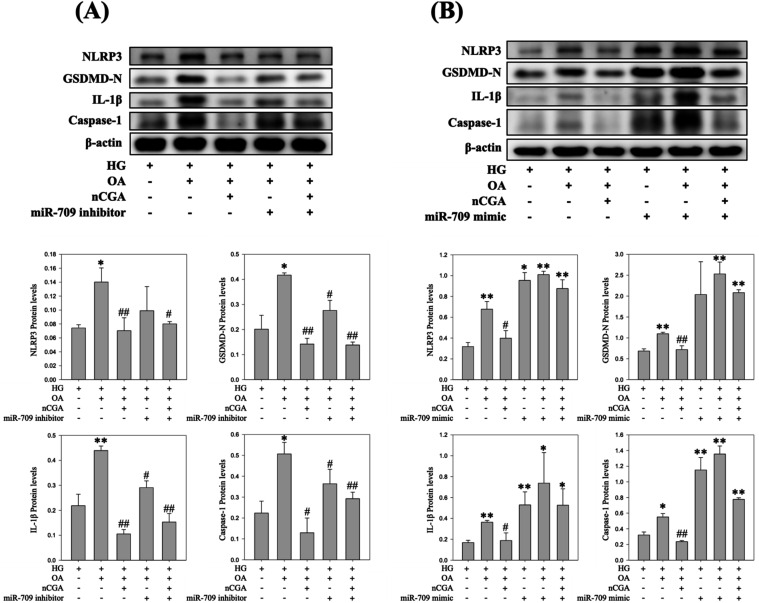
Effect of miR-709 inhibition and miR-709 overexpression on pyroptosis in glucolipotoxicity-induced MES-13 cells. (A) Western blot and quantitative analysis of NLRP3, GSDMD-N, IL-1β, and caspase-1 after treatment with HG, OA, nCGA, and miR-709 inhibitor. (B) Western blot and quantitative analysis of the same proteins after treatment with HG, OA, nCGA, and miR-709 mimic. Data are shown as mean ± SD *p < 0.05 compared to the HG group and #*p* < 0.05 compared to the HG + OA group.

## 4 Discussion

In this study, an integrative bioinformatics approach was employed to explore the molecular mechanisms underlying the renoprotective effects of neochlorogenic acid (nCGA). Through target mining from multiple databases (GeneCards, OMIM, TCMSP, and HERB), we identified 29 overlapping genes associated with inflammation, diabetic nephropathy (DN), and nCGA. Protein–protein interaction analysis revealed that NFKB1, TNF, and PTGS2 were the key hub genes, strongly suggesting that NF-κB signaling may be central to the effect of nCGA. Our findings indicate that nCGA plays a multifaceted protective role in renal cells, primarily through the modulation of oxidative stress, inflammatory pathways, and miRNA expression. Specifically, nCGA exerts a robust anti-inflammatory effect by inhibiting the NF-κB pathway, which is a key mediator of inflammation. This inhibition is evidenced by a reduction in the phosphorylation levels of NF-κB and its associated proteins, namely, IκB, which subsequently leads to a reduction in the levels of proinflammatory cytokines such as TNF-α, MCP-1, and ICAM-1. This dose-dependent reduction of these inflammatory markers underscores the efficacy of nCGA in mitigating inflammation-related cellular damage. Specifically, nCGA downregulates miR-709, which otherwise promotes ROS accumulation and inflammasome activation and upregulates miR-30a, which reduces NF-κB-mediated inflammation. Functional validation with mimics and inhibitors demonstrated that the modulation of these miRNAs is indispensable for the antipyroptotic effects of nCGA. Inhibition of miR-709 or overexpression of miR-30a suppressed pyroptotic mediators, while miR-709 mimic or miR-30a inhibitor reversed the inhibitory effect of nCGA. Collectively, these findings highlight a miRNA–inflammasome axis as a central mechanism by which nCGA mitigates glucolipotoxicity-induced injury in mesangial cells and protects against diabetic nephropathy.

We previously validated the beneficial effects of mulberry leaf extract containing nCGA in db/db mice.^17^ In this study, we indicated that nCGA inhibits NLRP3 inflammasome activation and its downstream pyroptotic proteins (e.g., GSDMD, caspase-1, and IL-1β) in MES-13 mesangial cells under glucolipotoxic conditions. This model mimics key pathological features of DN and allows us to better understand the underlying mechanisms of nCGA. Moreover, previous studies have also established that the initiation of pyroptosis involves the upregulation of NLRP3 and pro-IL-1β in response to various microbial or endogenous danger signals.^33^ Inflammation and pyroptosis are mechanistically interconnected processes that amplify renal injury in diabetic nephropathy. Inflammatory signaling, particularly through NF-κB activation, promotes the transcription of pro-IL-1β and NLRP3, thereby priming the inflammasome pathway.^34^ Once activated, the NLRP3 inflammasome triggers caspase-1 cleavage, which not only drives pyroptotic cell death through GSDMD pore formation but also facilitates the maturation and secretion of IL-1β and IL-18, further fueling inflammation.^35^ This creates a vicious cycle in which inflammation enhances pyroptosis, while pyroptosis exacerbates inflammation via the release of proinflammatory cytokines and damage-associated molecular patterns. Our findings demonstrate that nCGA disrupts this cycle by inhibiting both NF-κB-mediated inflammatory signaling and NLRP3 inflammasome activation, thereby attenuating inflammation-driven pyroptosis in mesangial cells. Notably, the generation of mitochondrial ROS is recognized as a critical upstream factor in the activation of the NLRP3 inflammasome, which subsequently triggers downstream pyroptotic processes.^36^ Our findings align with and extend these previous observations by demonstrating that nCGA significantly mitigates pyroptosis in MES-13 cells exposed to glucolipotoxicity. Specifically, treatment with nCGA reduced the expression levels of NLRP3, GSDMD, IL-1β, and caspase-1, key markers of pyroptotic activation. These results suggest that nCGA inhibits NLRP3 inflammasome activation and the subsequent cascade of pyroptotic events. Given the extensive evidence supporting mitochondrial ROS as a primary trigger for NLRP3 activation, the antioxidant properties of nCGA may play a crucial role in its inhibitory effects on pyroptosis.

Although both CGA and its isomer nCGA possess notable antioxidant and anti-inflammatory activities, emerging evidence suggests that nCGA may exert superior biological effects in certain pathological contexts due to its higher stability and distinct metabolic fate.^19^ For example, nCGA has shown enhanced modulation of the Nrf2 and miRNA signaling pathways in kidney and liver models, highlighting its potential as a more targeted therapeutic agent in metabolic inflammation.^17^ In addition, high glucose and lipid levels induce glucolipotoxicity, which is a common symptom of metabolic diseases. This condition leads to glomerular damage and adipose tissue dysfunction, particularly in patients with obesity, with the kidney being a key target organ for lipotoxicity-mediated damage.^11^ High glucose conditions contribute to oxidative stress in renal tissues by promoting excessive ROS generation and lipid peroxidation within glomerular cells.^37^ Mesangial expansion, characterized by the accumulation of extracellular matrix in the glomerulus, is a hallmark histopathological change observed in type 2 diabetes and obesity-related kidney disease.^4^ In the early stages of renal injury linked to obesity or prediabetes, common pathological features include albuminuria, glomerular hypertrophy, and expansion of the mesangial matrix.^11^ Our findings are consistent with those of previous studies, indicating that high glucose and lipid levels induce glucolipotoxicity, leading to glomerular damage in MES-13 cells. In addition, the protective effects of nCGA observed in our study are consistent with these established patterns, further validating the key role of oxidative stress and inflammation in DN.

Oxidized lipoproteins contribute significantly to renal injury by inducing oxidative stress and apoptosis in tubular epithelial cells through the recruitment of inflammatory monocytes and the upregulation of proinflammatory cytokines, thereby amplifying local inflammation and accelerating the progression of diabetic nephropathy.^11^ Previous studies have indicated that the activation of NF-κB and the transcription of specific proinflammatory chemokines in tubular epithelial cells are key markers of progressive DN.^38^ In our study, we observed that the inflammatory factors downstream of the NF-κB pathway, including TNF-α, MCP-1, and ICAM-1, were upregulated in the glucolipotoxicity (HG + OA) group. However, in the nCGA-treated (HG + OA + nCGA) group, the levels of these inflammatory markers were significantly reduced, with the extent of reduction positively correlating with the dose of nCGA.

In addition, the rationale for employing both miRNA mimics and inhibitors in this study was to provide bidirectional validation of the regulatory mechanisms. Mimics were used to overexpress the candidate miRNA and confirm whether the induced phenotype recapitulates the effects of nCGA, while inhibitors were applied to suppress the endogenous miRNA activity to test whether the protective effects are reversed. This dual strategy ensures that the modulation of NF-κB and Nrf2 signaling is specifically mediated by miR-30a and miR-709, minimizing the likelihood of off-target effects. Previous study successfully demonstrated gain- and loss-of-function validation using miRNA-424 mimics and inhibitors in synovial fibroblasts.^39^ These findings reinforce the appropriateness of our strategy in validating the role of miR-30a and miR-709 in DN. Furthermore, miRNAs are present in serum, urine, and other bodily fluids, making them highly sensitive indicators of changes within an organism. These molecules can be used in a noninvasive manner to monitor the diagnosis and progression of kidney diseases.^40^ In this context, our findings highlight that both miR-30a and miR-709, which are mechanistically involved in the regulation of NF-κB and Nrf2 pathways, may also serve as promising urinary biomarkers for the early detection and monitoring of diabetic nephropathy.^41^ The effect of nCGA on the expression of miRNA represents a novel mechanism with protective effects. In addition to our findings, accumulating evidence supports the anti-inflammatory function of miR-30a via the modulation of the NF-κB signaling pathway across various disease models. For instance, in IL-17-induced inflammatory responses, miR-30a-5p was shown to suppress NF-κB activation by directly targeting Act1, thereby reducing proinflammatory cytokine production in epithelial cells.^42^ Similarly, bovine colostrum-derived exosomal miR-30a-5p inhibited LPS-induced intestinal inflammation by downregulating TRAM and attenuating NF-κB signaling.^42^ These results highlight the conserved role of miR-30a in the regulation of innate immune pathways.

On the other hand, our findings reveal that miR-709 acts as a negative regulator of the antioxidant transcription factor Nrf2 while simultaneously promoting the expression of proinflammatory cytokines such as TNF-α. The use of a miR-709 mimic significantly downregulated Nrf2 expression, resulting in an increased accumulation of ROS and elevated levels of TNF-α. Conversely, the inhibition of miR-709 restored Nrf2 activity, reduced ROS levels, and suppressed the NF-κB/TNF-α signaling pathway, highlighting the pathological contribution of miR-709 to DN progression. One study reported that miR-709 impairs mitochondrial function and increases ROS levels in acute kidney injury.^43^ Another analysis identified miR-709 as a potential biomarker in diabetic nephropathy, with roles in both oxidative stress and inflammation.^16^ While past research emphasized its involvement in redox imbalance, this study extends these findings by showing that miR-709 also contributes to inflammatory cytokine expression. Its downregulation by neochlorogenic acid suggests a key role in modulating both the oxidative and inflammatory pathways in DN.

## 5 Conclusions

This study provides novel insights into the molecular mechanisms underlying the protective effects of nCGA against DN. It uniquely describes the dual regulatory role of nCGA in modulating the NF-κB and Nrf2 pathways through specific miRNAs. By elucidating how nCGA inhibits the NF-κB pathway through miR-30a and downregulates the expression of miR-709 to enhance Nrf2 signaling, we offer novel insights into the intricate balance between oxidative stress and inflammation in DN. This dual modulatory aspect of key molecular pathways positions our research as a pioneering contribution with the potential to considerably influence the development of innovative therapies for DN.
